# Comment on "Association between lipoprotein(a) and coronary heart disease risk in type 2 diabetes mellitus and evaluation of statin treatment effects"

**DOI:** 10.1590/1806-9282.20250418

**Published:** 2025-08-08

**Authors:** Abdullah Güner, Muhammet Hüseyin Erkan

**Affiliations:** 1 Konya City Hospital, Department of Cardiovascular Surgery – Konya, Türkiye.

Dear Editor,

We would like to share some thoughts on the study entitled "Association between lipoprotein(a) and coronary heart disease risk in type 2 diabetes mellitus and evaluation of statin treatment effects"^1^.

In this study, the severity of coronary artery disease (CAD) was only determined by coronary angiography, and patients were grouped on this basis. However, the use of angiography alone to assess disease severity may be limited. In particular, scoring systems such as the Syntax Score can more accurately quantify patients’ cardiovascular risk by more accurately assessing the degree of stenosis in the coronary artery and the extent of disease spread^2^. The Syntax Score plays a critical role in guiding the management of patients with multivessel disease, as higher scores usually indicate more complex disease and require more aggressive treatment. In addition, non-invasive modalities such as echocardiography are an important tool for determining disease severity and monitoring treatment response. In particular, stress echocardiography may allow earlier diagnosis and follow-up of CAD with echocardiographic findings including wall motion abnormalities as well as coronary flow assessment^3^. In this study, focusing only on angiographic findings may prevent a broader assessment of the clinical status of patients.

The assessment of lipid response seems to focus on the change between baseline and final lipid values. However, the study methodology does not clearly state whether lipid response was assessed based on the final value or the percentage change. The percentage change more objectively reflects the response to treatment, while the final value may only indicate treatment efficacy over a specific period. Percentage change allows for more accurate and meaningful comparisons of treatment response between patients, as those with higher baseline lipid levels may show a greater proportional response than those with lower baseline levels^4^. In addition, the section on lipid therapy highlighted the effect of statin treatment, but genetic differences and individual response to treatment were less addressed in the study. Genetic factors can have a significant impact on the efficacy of statins; for example, some individuals have a high response to statins, while others have a minimal response. This may be better understood through pharmacogenetic studies and may lead to personalized treatment protocols^5^.

In addition, the numerical differences between the study groups may indicate a potential problem with statistical power. The larger size of the severe CAD group may affect the analysis of this group's response to treatment, as results obtained in groups with small sample sizes may be less reliable. Such imbalances in numbers may make it difficult to compare treatment effects between groups and may lead to bias in statistical significance. It is necessary to check that the statistical methods of the trial can accurately account for these differences.

## Data Availability

The datasets generated and/or analyzed during the current study are available from the corresponding author upon reasonable request.
